# A practical guide to calculating vocal tract length and scale-invariant formant patterns

**DOI:** 10.3758/s13428-023-02288-x

**Published:** 2023-12-29

**Authors:** Andrey Anikin, Santiago Barreda, David Reby

**Affiliations:** 1 Division of Cognitive Science, Department of Philosophy, Lund University, Box 192, SE-221 00 Lund, Sweden; 2 ENES Bioacoustics Research Laboratory, CRNL Center for Research in Neuroscience in Lyon, University of Saint Étienne, 42023, St-Étienne, France; 3 Department of Linguistics, University of California, Davis, Davis, CA USA; 4 Institut Universitaire de France, 75005 Paris, France

**Keywords:** Formants, Speaker normalization, Vocal tract length normalization, Vowel, Body size

## Abstract

Formants (vocal tract resonances) are increasingly analyzed not only by phoneticians in speech but also by behavioral scientists studying diverse phenomena such as acoustic size exaggeration and articulatory abilities of non-human animals. This often involves estimating vocal tract length acoustically and producing scale-invariant representations of formant patterns. We present a theoretical framework and practical tools for carrying out this work, including open-source software solutions included in R packages *soundgen* and *phonTools*. Automatic formant measurement with linear predictive coding is error-prone, but *formant_app* provides an integrated environment for formant annotation and correction with visual and auditory feedback. Once measured, formants can be normalized using a single recording (intrinsic methods) or multiple recordings from the same individual (extrinsic methods). Intrinsic speaker normalization can be as simple as taking formant ratios and calculating the geometric mean as a measure of overall scale. The regression method implemented in the function *estimateVTL* calculates the apparent vocal tract length assuming a single-tube model, while its residuals provide a scale-invariant vowel space based on how far each formant deviates from equal spacing (the *schwa* function). Extrinsic speaker normalization provides more accurate estimates of speaker- and vowel-specific scale factors by pooling information across recordings with simple averaging or mixed models, which we illustrate with example datasets and R code. The take-home messages are to record several calls or vowels per individual, measure at least three or four formants, check formant measurements manually, treat uncertain values as missing, and use the statistical tools best suited to each modeling context.

Spectral manifestations of vocal tract resonances, known as formants, have long been analyzed in speech by linguists interested in acoustic differences between phonemes. The pattern formed by the first few formants, in particular, is largely responsible for the perceptual differences between vowels: when we hear the vowel in *heed* as different from *had*, it is largely the relative position of the first two formants, F1 and F2, that is responsible (Behrman, 2021; Johnson, 2011; Titze, 2000). Because longer vocal tracts have lower resonances, absolute values of formant frequencies depend on the size of the vocal tract and thus, indirectly, on the size of the speaker (Pisanski et al., 2014). In phonetics and automatic speech recognition, this creates a confound, and researchers often employ ‘normalization’ methods meant to segregate information related to the size of the speaker (i.e., vocal tract length, VTL) from information related to the linguistic content of the signal (i.e., the formant pattern). However, both types of information – formant pattern and VTL – are often of interest when investigating paralinguistic information. For example, speakers may manipulate their VTL in the context of acoustic body size exaggeration or dominance displays (Charlton & Reby, 2016; Pisanski et al., 2016b), and VTL estimates are often extracted from formants measured in speech (Belyk et al., 2022; Cartei et al., 2019) and in animal calls (Fitch, 1997; Pfefferle & Fischer, 2006; Reby et al., 2005; Reby & McComb, 2003). Formant patterns are also relevant in many non-speech vocalizations in relation to body size exaggeration (Pisanski et al., 2022) or the ability of non-human animals to articulate (Boë et al., 2017; Fitch et al., 2016).

Given the importance of formants as paralinguistic cues in nonverbal acoustic communication, formant analysis is increasingly performed outside phonetics and with different goals in mind. Instead of being a nuisance parameter, VTL is often the main measure of interest, and vocal tract normalization becomes both more challenging and more indispensable outside the human VTL range – for example, when articulatory abilities are compared in different animal species. While the available literature on speaker normalization is extensive, it is often highly technical, and there is no consensus about which of the variety of proposed methods are more applicable to particular research contexts, no simple guidelines, and few off-the-shelf tools for actually implementing the described algorithms. Above all, the literature on formant analysis and speaker normalization is written with human phonetic research in mind, and it does not necessarily address the needs of a researcher from other fields such as psychology or animal behavior. To fill this gap, in this paper we present up-to-date solutions for measuring and verifying formant frequencies, estimating VTL, and extracting size-invariant formant patterns with a particular focus on those situations in which an ‘easy’ solution is not available (i.e., when linguistic content cannot be perfectly controlled for).

All proposed software solutions are freely available and open-source; we also share well-documented R code needed to prepare the data and to fit the statistical models described in the text (supplements: https://osf.io/4c2r9/). In this paper, we focus on a few algorithms that are easy to implement, yet powerful and robust, and do not discuss methods that are now largely obsolete, such as calculating formant dispersion by averaging the spacings between adjacent formants (Fitch, 1997; Pfefferle & Fischer, 2006). An extensive comparison of historically described normalization algorithms can be found in the excellent recent review by Johnson and Sjerps (2021).

## Measuring formants

The first step in the analysis of both formant patterns and VTL is to measure formant frequencies. The standard algorithm for this task is linear predictive coding (LPC). LPC relies on the mathematics of z-transforms, but the principle may be easier to grasp as a simple autoregressive model predicting the signal from its past values (Fulop, 2011). Formants correspond to the delays at which the signal partially repeats itself as sound waves bounce back and forth in the vocal tract. At some wavelengths, constructive interference creates a resonance as the waveform and its echo align in phase. LPC finds the wavelengths at which constructive interference occurs (formant frequencies) and estimates the persistence of these echoes (formant bandwidths), wherein stronger and more long-lasting reflections correspond to formants with narrower bandwidths. A popular choice for performing LPC is *Praat* (Boersma, 2006), which provides both a graphical user interface and a scripting language for automatic batch processing of recordings. There are also *Praat* plugins intended to streamline formant analysis such as *Fast Track* (Barreda, 2021a). *Praat* returns estimates of the frequencies and bandwidths of individual formants, often F1 to F4. Working in R, LPC is implemented in the function *findformants* from the *phonTools* package (Barreda, 2015), and additional tools for vowel normalization are available in packages *vowels* (Kendall & Thomas, 2018) and *soundgen* (Anikin, 2019).

Unfortunately, the crucial first step of formant detection is error-prone because LPC estimates are biased towards strong harmonics, particularly when the fundamental frequency is high, and manual checks, if any, typically aim merely to exclude obviously incorrect measurements, rather than to correct this bias (Whalen et al., 2022). The level of accuracy of automatic LPC may be acceptable for many purposes in speech analysis, particularly when the algorithm is expertly fine-tuned and applied to carefully controlled stimuli such as steady vowels produced at low pitch and recorded in a noise-free environment. Unfortunately, the problem of bias and outright noise in LPC output is greatly exacerbated when analyzing high-pitched and noisy vocalizations recorded in real-life settings. Despite the ongoing search for better alternatives, manual verification and correction of automatic formant measurements remains the most reliable option (Whalen et al., 2022). To facilitate this task, we propose a new open-source software solution, *formant_app*, which is now included in the R package *soundgen* (Anikin, 2019).

**Fig. 1:**
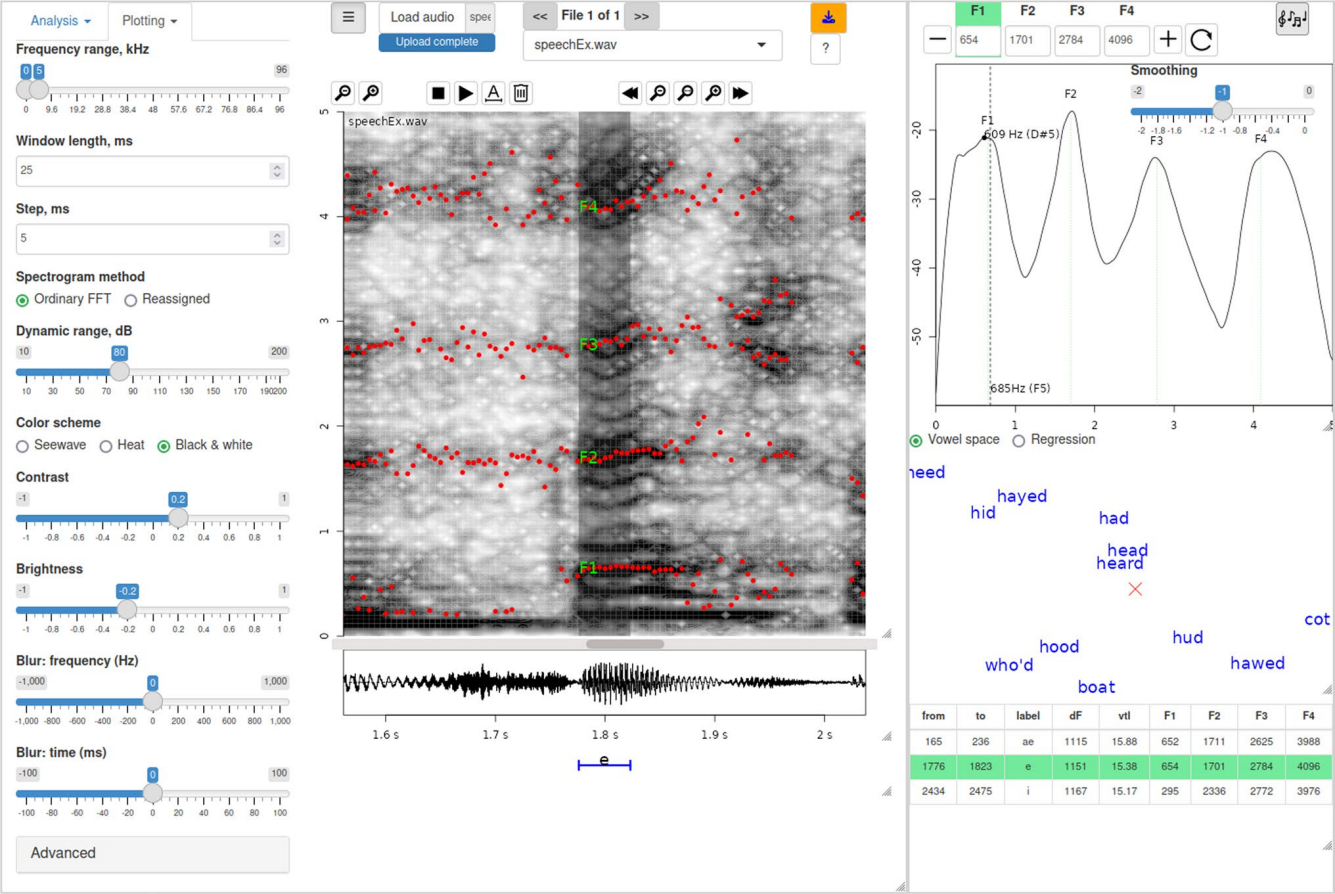
Software for verifying and adjusting LPC-based formant measurements: *soundgen::formant_app()*. This free, open-source web application runs in browsers Mozilla Firefox or Google Chrome and can be accessed online (https://cogsci.shinyapps.io/formant_app/) or locally by calling R function *formant_app()* from the *soundgen* package. The annotations can be made in the app and/or loaded in a csv file for checking together with the audio. The *red dots* and *yellow text labels* correspond to LPC estimates of formants F1 to F4 in the selected region, which can be verified visually and by listening to a synthetic vowel with the same formant frequencies, and then adjusted using the spectrogram or spectrum plot

When called from *RStudio* (RStudio Team, 2022) or from the bash terminal, the R function *formant_app()* opens an interactive web application, which runs LPC and offers tools for annotating, checking, and adjusting formant measurements with visual and auditory feedback (Fig.1). It is designed for finding and annotating one or more suitable vowel-like regions in each recording, obtaining LPC estimates of average formant frequencies in each region, and correcting them as needed. The main functional difference from *Praat* and its plugins is the focus on asserting the accuracy of average formant frequencies in each annotated region, rather than on exporting or correcting frame-by-frame formant tracks. This process is relatively fast and suitable for working with large datasets since a trained researcher can create and/or check about 100 annotated regions (hereafter, *tokens*) per hour. A brief description of the main settings, tools, and output of *formant_app* are provided below.

An essential, but commonly overlooked first step is to achieve a suitable visual representation of the analyzed sound by means of adjusting the spectrogram settings. This may seem too obvious to mention, but different tasks and different types of recordings require very different spectrogram representations, so the default settings will not suit all purposes; yet, manual verification is only as good as what we see (and hear – see below on auditory feedback). The conventional approach in phonetics is to use very short analysis windows, about 5 or 10 ms, so as to mask the harmonics and preserve rapid formant transitions, which are abundant and informative in speech. When analyzing non-speech material or focusing on VTL, however, it may be preferable to use much longer windows so as to see the harmonics of the fundamental frequency and ensure that the LPC contours are not locked to them, which is a very common problem in high-pitched vocalizations such as screams. Time- and frequency-reassigned spectrograms are also a useful visualization technique for formant analysis as this spectral representation, also available in *formant_app*, uses not only the magnitude, but also the phase of complex FFT in order to improve the time-frequency resolution (Fulop, 2011; Whalen et al., 2022). It may also be helpful to experiment with blurring or “unblurring” (sharpening) the spectrogram in time and/or frequency and to adjust the contrast and brightness settings.

Users can load one or several audio files, play them, and annotate the regions of interest. The audio can also be loaded together with an already prepared table of annotations in a single csv file (e.g., the output of an earlier session or a correctly formatted table with measurements from other sources such as *Praat*). For each annotated region, a new entry is added to the output table, which contains the start and end time, a manual label (which can be left blank), the frequencies of the first *n* formants, and the estimated VTL and formant spacing (dF). As explained in the next section, the principle behind these metrics is that, given a fixed articulation, proportional increases in the physical size of the vocal tract result in equal proportional increases in dF and estimated VTL.

The LPC analysis in *formant_app* is provided by the function *findformants* from R package *phonTools* (Barreda, 2015). The length of LPC window is independent of the FFT window length for plotting a spectrogram, but the principle is the same: a short window is preferable for tracking rapid formant transitions, while a long window is good for averaging out the noise in measurements and obtaining a robust long-term average value of each formant frequency. The number of LPC coefficients may be left blank, which defaults to two coefficients per kHz below the Nyquist frequency plus three extra coefficients, or set manually, which is particularly recommended for non-human vocalizations. Likewise, it may be necessary to adjust the minimum formant frequency and maximum formant bandwidth if working with sounds of animals much larger or smaller than humans – for example, elephant rumbles (Beeck et al., 2022). All these adjustments can improve the precision of automatic LPC considerably, but the main raison d’etre of *formant_app* is to facilitate manual correction of formant measurements.

There are four ways of doing this: having selected an annotation and the formant of interest with one of the formant buttons in the top right corner (Fig. 1), the user can single-click the spectrogram within the selected annotated region, single-click the spectrum of the selection, double-click the spectrum (in which case the formant frequency is set to the nearest spectral peak), or type in the new number in the text box within the formant button. Typing any non-numeric or empty string sets the formant to *NA*. In practice, it is most convenient to make adjustments using the spectrogram or spectrum, adjusting the amount of spectral smoothing with the provided slider. The panel underneath the spectrum provides other diagnostic plots: a speaker-normalized human vowel space based on Hillenbrand’s dataset (Hillenbrand et al., 1995) and the regression plots from the *estimateVTL* function. These plots are explained in detail in the next section.

Apart from the visual feedback provided by the spectrogram, spectrum, and diagnostic plots, it is helpful to hear the output – that is, to synthesize a sound with the measured formant frequencies and compare it to the original. Clicking the synthesis button in the top right corner of the app calls the *soundgen* function (Anikin, 2019), which creates and plays a synthetic vowel with the measured average formant frequencies in the current annotation. If a gross error is made, the difference is usually obvious to the ear. Because the pitch of the synthesized sound is adjusted in proportion to the apparent VTL implied by measured formants, it also provides a quick sanity check: for example, if an extra formant is detected or if one is skipped, the unrealistic VTL causes the sound to be very different in pitch from the preceding ones. The orange *export* button loads the complete table of annotations to R and writes it to disk as a plain-text csv file, which can be re-used in a later annotating session or analyzed statistically.

## Linear formants and VTL-based speaker normalization

**Fig. 2:**
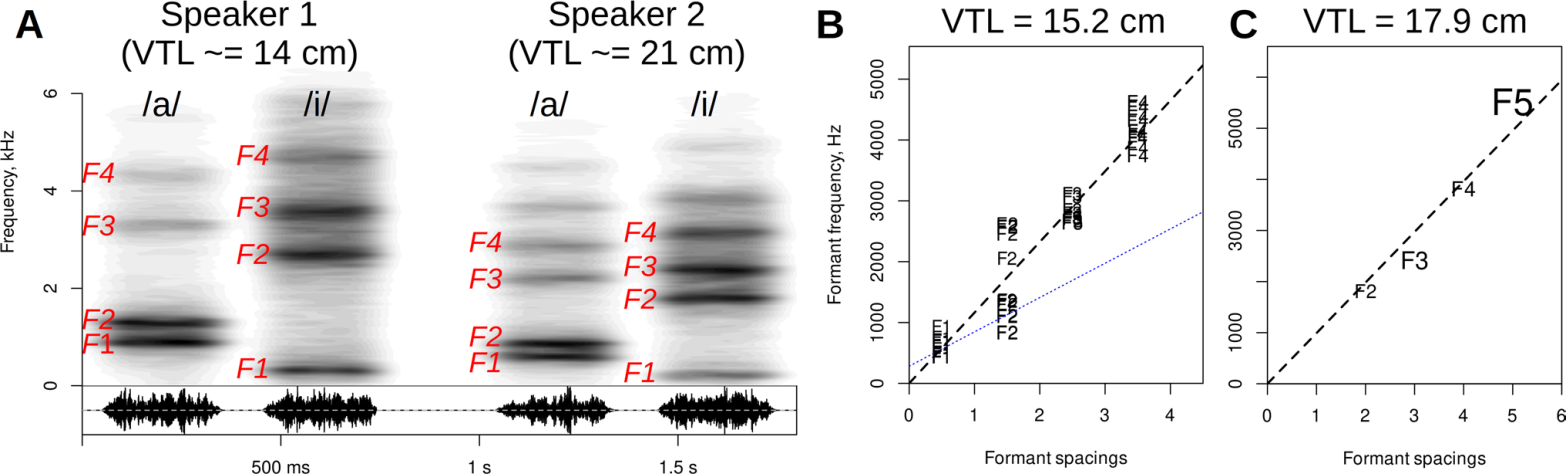
Estimation of vocal tract length from formants. **A** Conventional representation of formants on a linear frequency scale in spectrograms of synthetic vowels /a/ and /i/ by two speakers with exaggeratedly different VTLs: a female with apparent VTL of about 14 cm and a hypothetical very large man with a VTL of 21 cm (all formants scaled up by 50% compared to speaker 1). Note that, while the average formant spacing is smaller for speaker 2, the formant pattern is vowel-specific, although this fact is not visually obvious on a linear scale. The vowels were created with *soundgen* using noise as source and plotted with a 25-ms window and Gaussian blur of 75 ms in the time dimension to highlight the formants. **B** The output of the *estimateVTL* function applied to 12 vowels by a female speaker. The slope of the dashed regression line corresponds to formant spacing dF, and the *blue dotted line* shows where formants would be if they were integer multiples of F1 (as a precaution against LPC tracking harmonics instead of formants). **C** The output of *estimateVTL* applied to a single recording of a closed-mouth *mmm* by a male speaker. The size of formant labels shows their influence on VTL estimates (e.g., F5 is disproportionately important here; F1 is missing). The regression lines pass through 0 in both (B) and (C), but X coordinates of the formants differ because the vocal tract is modeled as a closed-open tube in (B) and a closed-closed tube in (C)

Once the formants are measured, it is time to see what their values tell us about the vocalizer’s vocal tract, especially its overall length (VTL) and articulatory configuration. Most people working with audio are used to seeing spectrograms on a linear frequency scale, where formants above F3 are approximately equidistant, just like harmonics. Deviations of the first two or three formants from this regular spacing are responsible for what we hear as different vowels. For example, /a/ has a relatively high F1 and low F2, whereas /i/ has a relatively low F1 and high F2 (Fig. 2A). To keep our terminology consistent, we refer to categories like /a/ and /i/ simply as *vowels*, and to continuous variation in formant patterns as *vowel quality*. Notably, the phenomenon is not restricted to speech: non-uniform formant patterns are also found outside human speech (Boë et al., 2017; Fitch et al., 2016), and they are also the result of articulatory changes in the shape of the vocal tract.

If the vocal tract is represented as a uniform tube closed at the larynx and open at the lips, it can be modeled as a quarter-wave resonator with formants found at fixed intervals (i.e., every dF Hz) starting from dF/2, so that F1 = 0.5 dF, F2 = 1.5dF, F3 = 2.5 dF, etc. If the vocal tract forms a closed-closed tube (i.e., closed at the glottis and at the mouth, as in a non-nasalized *mmm* produced with a closed velopharyngeal passage) or an open-open tube (i.e., open at the glottis and mouth, as during relaxed breathing) and if we ignore the effects of nasalization, it becomes a half-wave resonator with the same formant spacing, but now F1 = dF, F2 = 2 dF, F3 = 3 dF, and so on (Johnson, 2011; Titze, 2000). Obviously, no vocal tract is perfectly cylindrical, and the reality is often much more complicated because of articulation and the involvement of additional resonators such as the nasal cavity or air sacks (Beeck et al., 2022; Reby et al., 2016). Thus, a simple uniform-tube model often provides a reasonable first approximation to vocal tract resonances, but it is important to remember that the assumptions of this model become less and less tenable as the vocal tract deviates from a cylindrical shape.

Regardless of how the vocal tract is shaped, the average distance between formants, also known as formant spacing or dispersion (dF), is a linear function of VTL: a person with a 10% longer vocal tract in the same configuration will have formants that are 10% lower and 10% more closely spaced. Speakers have slightly different geometries of the vocal tract, so this scaling with VTL may not be precisely isometric (Fant, 1975), but most models assume that a single scaling constant suffices to describe the effect of VTL on all formants (Barreda, 2016; Barreda & Nearey, 2018; Turner et al., 2009), and uniform scaling may be a more appropriate model of human vowel perception (Barreda, 2021b). Of note, spreading or rounding the lips, moving the larynx, and changing the position of the tongue and mandible (Maeda & Laprie, 2013) may affect the VTL within speaker. Upper formants tend to be relatively more stable across vowels, and therefore constitute more reliable predictors of vocal tract size (Lammert & Narayanan, 2015; Wakita, 1977), but they also shift around during articulating, just as F4 rises in /i/ compared to /a/ in Fig. 2A. Thus, three factors affect formant frequencies: (1) speaker-typical VTL, (2) articulatory changes in VTL caused by rounding or pulling back the lips and moving the larynx, and (3) other vowel-specific articulatory changes in the shape of the vocal tract caused primarily by tongue and jaw movements. The first two factors affect all formants to various extents (e.g., lip rounding in humans lowers all resonances, but especially the resonance of the cavity in front of the tongue), while (3) primarily affects the lower two formants.

Because formants occur on average every dF in a single tube, dF can be estimated from the measured formant frequencies with simple averaging (Johnson, 2020) or linear regression (Reby & McComb, 2003) as long as the vocal tract is approximately cylindrical, as for central vowels such as the neutral schwa vowel /ə/. The regression method makes it straightforward to pool information across many recordings because the model can include any number of formants and tokens; it also has the further advantage of estimating both the most likely dF and its uncertainty. To diminish the influence of the highly variable lower formants on the estimated slope, the intercept can be set to zero, forcing the regression line to pass through origin. This modified regression method of VTL estimation was proposed by Reby and coauthors (Reby et al., 2005; Reby & McComb, 2003) and employed in many later studies (Belyk et al., 2022; Cartei et al., 2019). VTL is then calculated from dF as:

1 $$\mathrm{VTL}=\mathrm{speed}\ \mathrm{of}\ \mathrm{sound}/\left(2\times \mathrm{dF}\right)$$

The resulting measure is often referred to as eVTL for “estimated VTL”, but it might as well be called əVTL to emphasize its derivation from a single-tube, cylindrical model of the vocal tract and to distinguish it from the true anatomical VTL. A user-friendly implementation of this regression method is provided by the *soundgen* function *estimateVTL*, which accepts a vector of measured formant frequencies from a single token or a list of multiple formant frequencies from several tokens. Let us assume that we have measured formants F1 to F4 in 12 different vowels by the same speaker – for example, adult female “w_39” in the dataset by Hillenbrand et al. (1995) – and saved these values in a dataframe called *speaker1*:

$$\kern0.5em {\displaystyle \begin{array}{l}\mathrm{df}=\mathrm{read}.\mathrm{csv}\left({}^{\prime }../\mathrm{data}/\mathrm{hillenbrand}\_\mathrm{fmt}.{\mathrm{csv}}^{\prime },\mathrm{stringsAsFactors}=\mathrm{TRUE}\right)\\ {}\mathrm{speaker}1=\mathrm{df}\left[\mathrm{df}\$\mathrm{speaker}= =^{`}\mathrm{w}\_39,\right]\\ {}\mathrm{head}\left(\mathrm{speaker}1\right)\end{array}}\kern1.25em$$

**Table:** 

vowel	f1	f2	f3	f4
had	564	2442	NA	4038
cot	931	1348	2698	4540
hawed	752	1101	2616	3732
...etc. 12 vowels in total.				

The eVTL of this speaker, averaging across all 12 vowels, can be calculated as follows:

$${\displaystyle \begin{array}{c}\mathrm{estimateVTL}\Big(\mathrm{list}\left(\mathrm{f}1=\mathrm{speaker}1\$\mathrm{f}1,\mathrm{f}2=\mathrm{speaker}1\$\mathrm{f}2,\mathrm{f}3=\mathrm{speaker}1\$\mathrm{f}3,\mathrm{f}4=\mathrm{speaker}1\$\mathrm{f}4\right),\\ {}\mathrm{tube}={}^{\prime}\mathrm{closed}-{\mathrm{open}}^{\prime },\mathrm{output}={}^{\prime }{\mathrm{detailed}}^{\prime}\Big)\#\kern0.5em\mathrm{eVTL}=15.2\ \mathrm{cm},95\%\mathrm{CI}\ \left[14.5,16.0\right]\end{array}}$$

Note that the point estimate of eVTL is the same whether we enter all 12 vowels individually or only the average frequency of each formant across these vowels, but its precision (estimated from the standard error in the linear regression model for dF as a function of observed formant frequencies) changes and becomes less meaningful when averaging formant frequencies before the regression because we lose the information about the dispersion of each formant around the regression line. Compare:

$$\kern1em {\displaystyle \begin{array}{c}\mathrm{means}=\mathrm{colMeans}\left(\mathrm{speaker}1\left[,\mathrm{c}\left({}^{`}\mathrm{f}{1}^{'},{}^{`}\mathrm{f}{2}^{'},{}^{`}\mathrm{f}{3}^{'},{}^{`}\mathrm{f}{4}^{'}\right)\right],\mathrm{na}.\mathrm{rm}=\mathrm{TRUE}\right)\kern1em \#\kern1em567\ 1672\ 2789\ 4171\\ {}\mathrm{estimateVTL}\left(\mathrm{means},\mathrm{tube}={}^{\prime}\mathrm{closed}-{\mathrm{open}}^{\prime },\mathrm{output} =^{`}{\mathrm{detailed}}^{'}\right)\kern0.5em \#\kern0.5em \mathrm{eVTL}=15.2\ \mathrm{cm},95\%\mathrm{CI}\ \left[14.7,15.8\right]\end{array}}$$

**Fig. 3:**
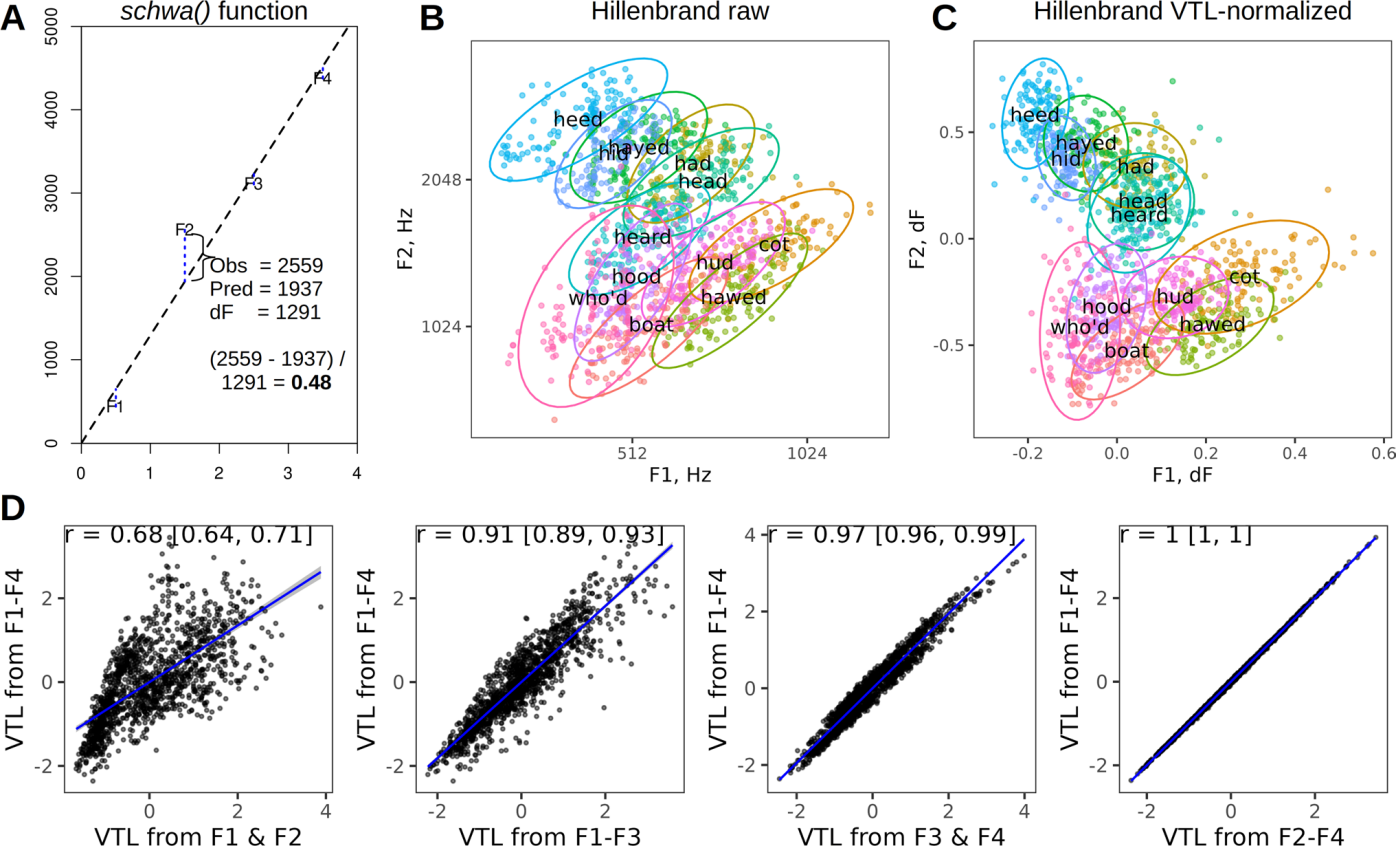
Speaker normalization based on eVTL. **A** The algorithm for calculating VTL-normalized formant frequencies as residuals from the regression line fit to observed formants in /i/ by an adult female. F2 is 0.48 dF units above the regression line, so normalized F2 = 0.48. **B** The original formant frequencies per token in (Hillenbrand et al., 1995). There is a lot of overlap between vowels, and vowel clusters are not clearly defined because the sample includes men, women, and children. **C** Vowels form more compact clusters in the space of VTL-normalized formants. The point (0, 0) corresponds to equidistant formants, as in a cylindrical tube or central vowels such as the *schwa* /ә/. **D** eVTL is primarily driven by upper formants: scatterplots and Pearson’s correlations (with 95% CI) between eVTL values calculated from a single token with different combinations of formants measured

As before, the point estimate of eVTL is 15.2 cm, but the confidence interval is now different. As shown in Fig. 3B, the slope of regression line (dF) is estimated at 1162 Hz, which corresponds to a VTL of 15.2 cm according to Eq. (1). Missing values marked *NA* do not cause the entire token to be dropped because each formant value is used as an independent point in the regression model. Likewise, eVTL can be calculated from a single token using any combination of measured and missing formants: the only requirement is that the index of each formant should be correct. Here is an example of a closed-mouth vocalization with unknown F1 (Fig. 3C):

$$\mathrm{estimateVTL}\left(\mathrm{c}\left(\mathrm{NA},1800,2400,3800,5500\right),\mathrm{tube}={}^{\prime}\mathrm{closed}-{\mathrm{closed}}^{\prime}\right)\kern0.5em \#\kern0.5em17.9\kern0.5em\mathrm{cm}$$

An alternative is to relax the assumptions of the model and evaluate both slope and intercept. For example, we can omit the missing F1 in the example above and not set the intercept to zero (the *tube* argument is removed because it makes no difference in models that do not set the intercept to zero):

$$\mathrm{estimateVTL}\left(\mathrm{c}\left(1800,2400,3800,5500\right),\mathrm{interceptZero}=\mathrm{FALSE}\right)\kern0.5em \#\kern0.5em14.2\ \mathrm{cm}$$

Note that we can only drop leading *NA*s: we still need to make sure we do not miss a formant in the middle: for example, we cannot omit the *NA* in “c(1800, NA, 2400)”, otherwise the estimated formant spacing will be incorrect. Furthermore, not setting the intercept to zero makes eVTL more sensitive to vowel-specific variation in the lower formants. In general, it is thus preferable to use the correct formant indices without omitting the *NAs* and to set the intercept to zero for the most accurate VTL estimates. To calculate eVTL for each token – each row in dataframe *df* similar to the dataset *speaker1* shown above – we call *estimateVTL* for each row:

$$\mathrm{for}\ \left(\mathrm{i}\ \mathrm{in}\ \text{1:}\mathrm{nrow}\left(\mathrm{df}\right)\right)\ \mathrm{df}\$\mathrm{vtl}\left[\mathrm{i}\right]=\mathrm{estimateVTL}\left(\mathrm{as}.\mathrm{numeric}\left(\mathrm{df}\left[\mathrm{i},\mathrm{c}\left({}^{\prime}\mathrm{f}{1}^{\prime },{}^{\prime}\mathrm{f}{2}^{\prime },{}^{\prime}\mathrm{f}{3}^{\prime },{}^{\prime}\mathrm{f}{4}^{\prime}\right)\right]\right)\right)$$

Alternatively, if we need a single eVTL estimate per speaker, we set up a dataframe with speaker names and call *estimateVTL* once per speaker:

$${\displaystyle \begin{array}{l}\mathrm{sp}=\mathrm{data}.\mathrm{frame}\left(\mathrm{sp}\mathrm{eaker}=\mathrm{unique}\left(\mathrm{df}\$\mathrm{speaker}\right)\right)\\ {}\mathrm{for}\ \left(\mathrm{i}\ \mathrm{in}\ \text{1:}\mathrm{nrow}\left(\mathrm{sp}\right)\right)\ \small\{\\ {}\begin{array}{l}\kern.5em\#\kern.5em\mathrm{subset}\ \mathrm{the}\ \mathrm{dataframe},\ \mathrm{selecting}\ \mathrm{one}\ \mathrm{power}\\\kern0.5em \mathrm{temp}=\mathrm{df}\left[\mathrm{df}\$\mathrm{speaker}==\mathrm{sp}\$\mathrm{speaker}\left[\mathrm{i}\right],\right]\\ {}\kern0.5em \#\kern0.5em\mathrm{estimate}\ \mathrm{VTL}\ \mathrm{for}\ \mathrm{this}\ \mathrm{speaker}\\ {}\begin{array}{l}\kern0.3em \mathrm{sp}\$\mathrm{vtl}\left[\mathrm{i}\right]=\mathrm{estimateVTL}\left(\mathrm{list}\left(\mathrm{f}1=\mathrm{temp}\$\mathrm{f}1,\right.\right.\\{}\left.\left.\kern.9em\mathrm{f}2=\mathrm{temp}\$\mathrm{f}2,\mathrm{f}3=\mathrm{temp}\$\mathrm{f}3,\mathrm{f}4=\mathrm{temp}\$\mathrm{f}4\right)\right)\\ {}\small\}\end{array}\end{array}\end{array}}$$

A nice bonus feature of the regression method of VTL estimation is that dF-normalized residuals – distances from the observed formants to the regression line – provide a scale-invariant measure of the formant pattern (or vowel quality, in the case of human speech). In a perfectly cylindrical vocal tract, each formant should fall exactly on the regression line, which approximately corresponds to central vowels such as the *schwa* /ә/. In reality, the observed formants tend to deviate from equal spacing, and thus from the regression line. The residual of each formant, normalized by dividing it by formant spacing dF, gives relative formant frequencies relative to the resonances of a uniform tube of the same length (Fig. 3A). The residuals can also be expressed in semitones, but then their variance decreases from lower to upper formants because higher formants are more closely spaced on a logarithmic frequency scale. In contrast, residuals expressed in dF units are independent of both formant index and the overall scale – thus, multiplying all formants by the same scale factor has no effect on the normalized formant frequencies. As a result, the clusters corresponding to different vowels spoken by children, women, and men in Hillenbrand’s dataset become visually more compact after eVTL normalization compared to the original measurements in Hz (Fig. 3B, C), which will be proven more formally when we compare different methods of vowel normalization below. Likewise, deviations from equal spacing in dF units can be used to normalize formant frequencies in open-mouth vocalizations of animals of any size, from kittens to elephants, making it possible to project them onto the same vocal-tract normalized space. Moreover, the resulting space can be directly juxtaposed with the familiar space of human vowels for comparison, as in the diagnostic plot of vowel space in *formant_app* (Fig. 1).

A convenient way to calculate VTL-normalized formant frequencies is to call the function *schwa()* from the *soundgen* package. For example, the vowel /i/ in Fig. 3A can be normalized by running:

$$\mathrm{schwa}\left(\mathrm{c}\left(436,2559,3104,4375\right),\mathrm{plot}=\mathrm{TRUE}\right)$$

This returns the estimated dF (1291 Hz), eVTL (13.7 cm), predicted frequencies of F1–F4 in a *schwa* vowel for this eVTL, and the deviation of each measured formant from the schwa (– 0.16, 0.48, – 0.09, and – 0.11 dF units for F1–F4, respectively), together with a plot of normalized vowel centroids from Hillenbrand’s dataset as a visual reference framework. Thus, we see that F1 is relatively low in /i/, whereas F2 is high relative to where it would be in a cylindrical vocal tract of the same length as the eVTL estimated from these formant frequencies. To normalize all formants in dataframe *df*, we call the *schwa* function for every row, just as we did with *estimateVTL*:

$${\displaystyle \begin{array}{l}\mathrm{f}\mathrm{or}\ \left(\mathrm{i}\ \mathrm{in}\ \text{1:}\mathrm{nrow}\left(\mathrm{df}\right)\right)\ \small\{\\ {} \mathrm{schwa}\_\mathrm{i}=\mathrm{schwa}\left(\mathrm{as}.\mathrm{numeric}\left(\mathrm{df}\left[\mathrm{i},\mathrm{c}\left({}^\text`\mathrm{f}{1}^\text',{}^\text`\mathrm{f}{2}^\text',{}^\text`\mathrm{f}{3}^\text',{}^\text`\mathrm{f}{4}^\text'\right)\right]\right)\right)\\ {}\begin{array}{c}\kern-15em \mathrm{df}\left[\mathrm{i},\mathrm{c}\left({}^\text`\mathrm{f}1{\mathrm{rel}}^\text',{}^\text`\mathrm{f}2{\mathrm{rel}}^\text',{}^\text`\mathrm{f}3{\mathrm{rel}}^\text',{}^\text`\mathrm{f}4{\mathrm{rel}}^\text'\right)\right]{=}\\\kern-18em\mathrm{schwa}\_\mathrm{i}\$\mathrm{f}\mathrm{f}\_{\text{relative}\_}{\mathrm{dF}}\kern3em \\ {}\kern-10.6em \#\kern0.2em\mathrm{we}\ \mathrm{can}\ \mathrm{also}\ \mathrm{save}\ \mathrm{VTL}\ \mathrm{here},\mathrm{without}\ \mathrm{having}\ \\\kern-21.6em\#\kern0.2em\mathrm{to}\kern0.2em\mathrm{call}\ \mathrm{estimateVTL}\left(\right)\\ {}\begin{array}{l}\kern-.5em\mathrm{df}\$\mathrm{vtl}\left[\mathrm{i}\right]=\mathrm{schwa}\_\mathrm{i}\$\mathrm{vtl}\_\mathrm{apparent}\\ {}\small\}\end{array}\kern16.25em \end{array}\end{array}}$$

As mentioned above, the regression method of VTL estimation and algorithmic normalization can handle missing values: the regression line can be fit using any number and combination of formant frequencies as long as each measured value is assigned to the correct formant index. Missing values do not need to be excluded before executing the code above, although the corresponding normalized values will likewise be missing (*NA*). Moreover, different combinations of formants (F1 to F3, F3 and F5, etc.) can be measured in different recordings pooled to provide a single estimate of the speaker-typical eVTL. An important special case is systematic omission of some formants. For example, sometimes it is impossible to measure formants F1 and F2 because of background noise or high fundamental frequency; on other occasions, upper formants may be invisible because the signal is weak, the voice is breathy and quiet, or the sampling rate is too low to encode high-frequency formants, as in old recordings. As shown in Fig. 3D, eVTL estimated with the intercept fixed at zero is barely affected by F1, and even only F3 and F4 produce VTL estimates that are correlated with estimates from F1–F4 with Pearson’s *r* = .97. Thus, if the VTL is the measure of interest and F1–F2 cannot be measured reliably, we can safely treat them as missing and still estimate eVTL from a few upper formants. In contrast, using only the lower formants, especially just the first two, produces highly unstable eVTL estimates (Fig. 3D).

In sum, the method of estimating the apparent VTL from regression-derived formant spacing dF provides a simple and intuitive metric of the overall vocal tract size, while dF-normalized residuals constitute a scale-invariant measure of vowel quality. Unfortunately, as noted above, this method is limited by the often unrealistic assumption that the vocal tract is nearly cylindrical. In actual fact, many human vowels are articulated with tongue positions that fundamentally violate the assumptions of a uniform-tube model (Johnson, 2011). Therefore, it is crucial to emphasize that eVTL is not the same as anatomical VTL, and it is most meaningful to compare eVTL in vocalizations that have either schwa-like or at least the same vowel quality (or the same set of vowel qualities). For example, we can compare eVTL in two /a/-like vocalizations by the same or two different individuals, but we cannot meaningfully compare vocal tract length based on eVTL in two different vowels.

## Logarithmic formants and speaker normalization by transposition

It is convenient to represent human formants on a linear frequency scale when the focus is on voice production because vocal tract resonances occur approximately every dF Hz. However, our auditory perception is approximately logarithmic in the relevant frequency range (Fastl & Zwicker, 2006). Furthermore, the invariance in formant ratios between speakers saying the same vowel becomes more obvious if these ratios are log-transformed – that is, if we convert ratios to musical intervals. Using base-two logarithms, we obtain the conventional musical scale of octaves or semitones; for instance, if F1 = 500 Hz and F2 = 1500 Hz, they form an interval of 19 semitones, or an octave and pure fifth:

2 $$\mathit{\log}2\left(F2/F1\right)=\mathit{\log}2(F2)-\mathit{\log}2(F1)=\mathit{\log}2(1500)-\mathit{\log}2(500)\sim =1.58\ octaves, or\ 1.58\times 12=19\ semitones$$

**Fig. 4:**
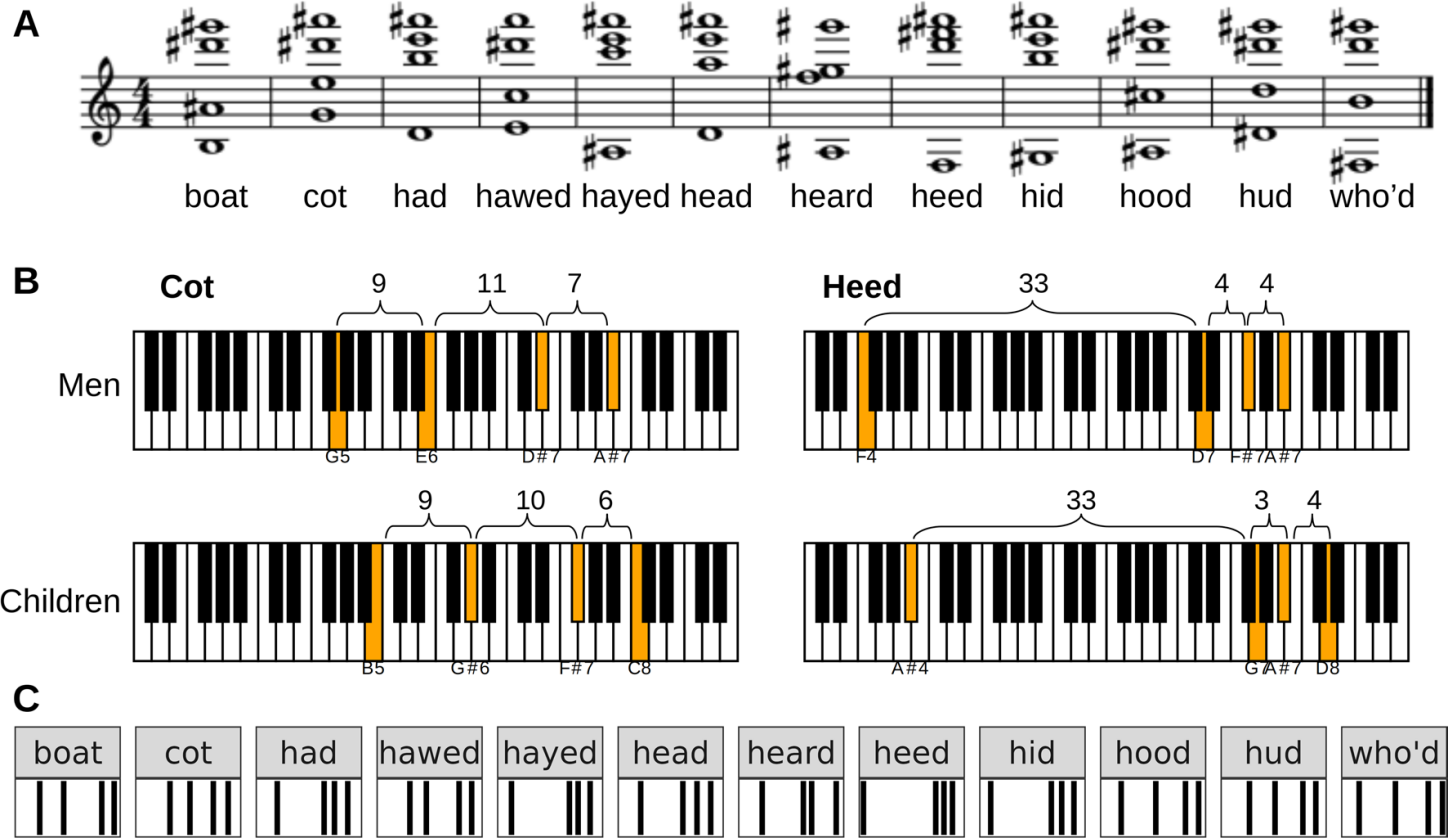
Representations of formants on a logarithmic frequency scale. **A** Formants as musical chords: 12 vowels by an average adult man in Hillenbrand’s dataset shown as musical chords, where each note corresponds to a formant (transposed down by one octave). **B** Vowels /ɑ/ and /i:/ by an average adult man and child, with formants shown as notes on a piano keyboard (an octave consists of 12 notes separated by one semitone). The highlighted notes correspond to formants F1 to F4, with the interval between them marked in semitones. The children’s formants are transposed up by 4 or 5 semitones compared to adult men, but the intervals between formants are nearly identical. **C** Formants as bar codes: log-formants form distinct, vowel-dependent but VTL-invariant patterns. Note that this is only the case if we assume that all formants scale uniformly with VTL, and only on a logarithmic scale because the spacing between adjacent formants measured in Hz varies with VTL

If we then take log-ratios of F2 to F1, F3 to F2, F4 to F3, and so on, each vowel is transformed into a musical chord composed of formants instead of notes (Fig. 4A). Intervals between formants can also be expressed in quasi-logarithmic perceptual units such as mels or barks instead of semitones (Syrdal & Gopal, 1986). The key insight is that the chord formed by each vowel is very similar across speakers, regardless of the size of their vocal tracts. This is known as the ‘uniform scaling’ or ‘constant ratio’ hypothesis, and it is a very old observation, first published in late 19th century and then repeatedly proposed with slight variations by new generations of researchers (Johnson & Sjerps, 2021; Miller, 1989). When formants are reconceptualized as musical chords, it is immediately obvious how they might be normalized across speakers: all we need to do is shift, or transpose, the chords to some standard reference point (Fig. 4B). For instance, we can subtract the average log-frequency, which is mathematically equivalent to dividing each formant F_n_ on the linear scale (Hz) by the geometric mean of all measured formants. For instance, working with formants F1 to F4:

3 $$\mathit{\log}2\left( geometric\ mean\right)=\mathit{\log}2\left({\left(F1\ \times\ F2\ \times\ F3\ \times\ F4\right)}^{1/4}\right)=\left(\mathit{\log}2(F1)+\mathit{\log}2(F2)+\mathit{\log}2(F3)+\mathit{\log}2(F4)\right)/4= mean\left(\mathit{\log}- formant\right)$$

4 $$\mathrm{l} og2\left({F}_n/ geometric\ mean\right)=\mathit{\log}2\left({F}_n\right)\hbox{--} \mathit{\log}2\left( geometric\ mean\right)=\mathit{\log}2\left({F}_n\right)\hbox{--} mean\left(\mathit{\log}- formant\right)$$

This method is known in phonetics as log-mean (or Nearey) normalization (Nearey, 1978) or the sliding-template model (Nearey & Assmann, 2007). An R implementation is available in the function *normalize* from the *phonTools* package (Barreda, 2015). Transposing formant patterns does not add any new information about the vowel. However, if we are also interested in the size of the vocal tract, the mean log-formant provides a ready-made scale metric. Simply put, the musical chord created by the formants depends on the shape of the vocal tract and thus encodes the vowel, while the chord’s location along the frequency scale (analogous to a piano keyboard) depends on the length of the vocal tract and thus conveys some information about the speaker’s size (Turner et al., 2009).

Linear normalization with *estimateVTL* converts any number of formants, and any combination of missing and measured values, to the same metric of eVTL measured in cm. In contrast, the mean log-frequency requires that the same number of formants be measured in all tokens, with no missing values, otherwise scale estimates will not be commensurate across tokens. Fortunately, this limitation can be overcome by using a statistical approach if multiple tokens are recorded per speaker: each measured formant frequency can be modeled as a function of formant index, vowel, experimental condition, and other relevant predictors, including speaker-specific scale constants, as suggested by Barreda and Nearey (2018). Their regression approach can be updated to take advantage of the flexibility and rich information provided by multilevel Bayesian models. For instance, consider the situation where we are interested in estimating a speaker-specific scaling factor (let us call it *k*) in Hillenbrand’s dataset. We fit a multilevel Bayesian model predicting log-transformed formant frequency using the *brms* package in R (Bürkner, 2017). Assuming the data is in the ‘long’ format (one row = one formant), model K1 can be written as:

$${\displaystyle \begin{array}{l}\operatorname{mod}\_\mathrm{K}1=\mathrm{brm}\left(\mathrm{bf}\right(\\ {}\kern1em \log \_\mathrm{frequency}\sim \mathrm{formant}\_\mathrm{index}+\mathrm{vowel}+\mathrm{formant}\_\mathrm{index}:\mathrm{vowel}+\left(1|\mathrm{speaker}\right),\\ {}\kern1em \mathrm{sigma}\sim \mathrm{formant}\_\mathrm{index}\left),\dots \right)\end{array}}$$

The first line of this formula specifies that the measured log-frequency depends on formant number (main effect of *formant_index*), overall vowel-specific scale factor (main effect of *vowel*), and an interaction between these two variables, which means that different formants can shift around independently in different vowels. The second line specifies that each log-formant can have a different standard deviation, which is clearly the case both on a linear scale (because the lower formants are much more variable than the upper ones, at least in human vowels) and even more so on a logarithmic scale (because upper formants are increasingly closely spaced). This may seem like a trivial point, but the omission of this part can have a dramatic effect on results: as demonstrated in the example in supplements (*scenario2.html*), a model with the same SD for all formants makes predictions very similar to mean log-frequency, whereas a model with different SDs puts more weight on the less variable upper formants, behaving more similarly to eVTL.

The main measure of interest in model K1 is each speaker’s ‘random’ intercept, or scale factor *k*, which shows how much higher or lower formant log-frequency is on average (across all tokens, formants, and vowels) in a specific person compared to the average person in the sample. The unit of *k* is binary-logarithmic, so a *k* of 1 means that a speaker has formants twice as high in frequency as the population average. Missing values of some formants do not require dropping the entire token because the unit of analysis is a single formant (Barreda & Nearey, 2018). For ease of interpretation, *k* can be converted into a measure of relative VTL, which we refer to as kVTL, by taking some reference VTL value (e.g., 17 cm or, more meaningfully, the mean eVTL in our sample) and dividing it by 2^*k*:

$$\mathrm{df}\$\mathrm{k}\mathrm{VTL}=\mathrm{mean}\left(\mathrm{df}\$\mathrm{vtl}\right)/\left(2\hat{\phantom{0}} \mathrm{df}\$\mathrm{k}\right)$$

Thus, for each grouping level (vowel, speaker, condition, etc.), kVTL can be calculated from the corresponding scale factor *k* in relation to some standard reference value. It must be emphasized that, although kVTL is measured in centimeters, it is not an absolute anatomical measure but merely a projection of *k* onto a more intuitive scale. Likewise, the formant frequencies themselves are commonly normalized by dividing them by some scale normalization factor. For instance, if we use mean log-formant as our measure of scale, its sample mean provides a reference point for normalizing formant frequencies, as follows:

$${\displaystyle \begin{array}{l}\mathrm{correction}=2\hat{\phantom{0}} \mathrm{df}\$\mathrm{mean}\_\mathrm{logF}/2\hat{\phantom{0}} \mathrm{mean}\left(\mathrm{df}\$\mathrm{mean}\_\mathrm{logF}\right)\\ {}\mathrm{df}\left[,\mathrm{c}\left({}^{`}\mathrm{F}{1}^{'},{}^{`}\mathrm{F}{2}^{'},{}^{`}\mathrm{F}{3}^{'},{}^{`}\mathrm{F}{4}^{'}\right)\right]=\mathrm{df}\left[,\mathrm{c}\left({}^{`}\mathrm{F}{1}^{'},{}^{`}\mathrm{F}{2}^{'},{}^{`}\mathrm{F}{3}^{'},{}^{`}\mathrm{F}{4}^{'}\right)\right]/\mathrm{correction}\kern0.5em \#\,\mathrm{normalized}\ \mathrm{formants}\ \mathrm{in}\ \mathrm{Hz}\end{array}}$$

The difference from the *schwa* normalization in Fig. 4 is that we preserve the natural scale: formant frequencies are still expressed in Hz, but now they are normalized to remove variation in overall scale between speakers – in this case, the log-formant of each token becomes equal to the global mean log-formant. Likewise, observed formants in each vowel can be normalized by *k*:

$$\begin{aligned}{\displaystyle \begin{array}{l}\mathrm{correction}=2\hat{\phantom{0}} \mathrm{df}\$\mathrm{k}\\\#\ \mathrm{technically},2\hat{\phantom{0}} \left(\mathrm{df}\$\mathrm{k}\hbox{--} \mathrm{mean}\left(\mathrm{df}\$\mathrm{k}\right)\right),\\\#\ \mathrm{but}\ \mathrm{the}\ \mathrm{mean}\ \mathrm{of}\ \mathrm{k}\ \mathrm{approaches}\ 0\\ {}\mathrm{df}\left[,\mathrm{c}\left({}^\text`\mathrm{F}{1}^\text`,{}^\text`\mathrm{F}{2}^\text`,{}^\text`\mathrm{F}{3}^\text`,{}^\text`\mathrm{F}{4}^\text`\right)\right]=\\\mathrm{df}\left[,\mathrm{c}\left({}^\text`\mathrm{F}{1}^\text`,{}^\text`\mathrm{F}{2}^\text`,{}^\text`\mathrm{F}{3}^\text`,{}^\text`\mathrm{F}{4}^\text`\right)\right]/\mathrm{correction}\\ \#\ \mathrm{normalized}\ \mathrm{formants}\ \mathrm{in}\ \mathrm{Hz}\end{array}}\end{aligned}$$

To conclude this brief introduction to the technique of using mixed models to model scale factor *k*, its crucial statistical advantage is that the effect of interest (e.g., differences in VTL between vowels or experimental conditions) can be modeled directly, producing realistic confidence intervals. In contrast, raw mean log-formant cannot deal with missing formant values, while estimating eVTL first and then comparing it across vowels or conditions typically fails to take into account the uncertainty in eVTL itself, which is in fact only a point-estimate of a model parameter inferred with some uncertainty. Despite these advantages of the proposed modeling method, it is important to remember that there is currently no silver-bullet model that would produce accurate estimates of the true anatomical VTL from a single vocalization of an unknown vowel using formant frequencies alone. We discuss what makes this task so challenging in the next section.

## A comparison of methods for estimating VTL

To sum up the discussion so far, two pieces of information are available from measured formant frequencies. One is the acoustically estimated or “apparent” VTL (an approximation of the anatomical VTL), which is a function of the average spacing between formants on a linear scale, or of their average location on a logarithmic scale. The other is the formant pattern, perceived as vowel quality in sounds resembling human vowels, which depends on the ratios or musical intervals between the first two or three formants.

Formant analysis would be much easier if formant patterns and VTL were fully independent, but they are not. As already mentioned, articulatory movements can lengthen or shorten the vocal tract, so that a person saying /u/ may have a longer vocal tract (and a different scaling constant calculated from this one recording) compared to the same person saying /i/. Furthermore, in the absence of detectable higher formants the task of estimating both size and vowel quality suffers from circularity because each one depends on the other: the same absolute frequencies of F1 and F2 can correspond to different vowels depending on VTL, and different VTLs can be inferred depending on which vowel is perceived (Barreda, 2020). The same absolute formant frequencies can also result from a variety of vocal tract configurations and lengths (Atal et al., 1978). Thus, an observed pattern of formant frequencies may be compatible not with one, but with a whole range of VTLs, depending on the speaker’s vocal tract anatomy and manner of articulation.

The estimation of both VTL and vowel quality can improve dramatically if we have several vowels from the same speaker (Johnson & Sjerps, 2021), as opposed to estimates based on a single category. Using information extrinsic (i.e., external) to a particular analyzed token is known as *extrinsic* normalization, and it results in what we will call ‘multiple’ estimates of VTL. These can be contrasted with ‘single-shot’ estimates based only on the information *intrinsic* to each token.

Keeping this distinction in mind, we can compare the performance of different normalization algorithms discussed above and evaluate the validity of different VTL estimates, of which we have discussed three: one-shot or multiple eVTL, one-shot or multiple mean log-formant, and multiple scale factor *k* estimated with mixed model K1. Unfortunately, the ground truth of anatomical VTL is not available for Hillenbrand’s or other comparable datasets, and there are apparently no suitable banks of recordings with anatomical measurements of actual VTL as well as formant frequencies. Imaging techniques (magnetic resonance or X-ray) were used in several studies to study within-subject changes in anatomical VTL during speech, but with too few speakers (Lammert & Narayanan, 2015; Maeda & Laprie, 2013) and usually without recording any audio (Belyk et al., 2022; Fitch & Giedd, 1999; Kim et al., 2020).

**Fig. 5:**
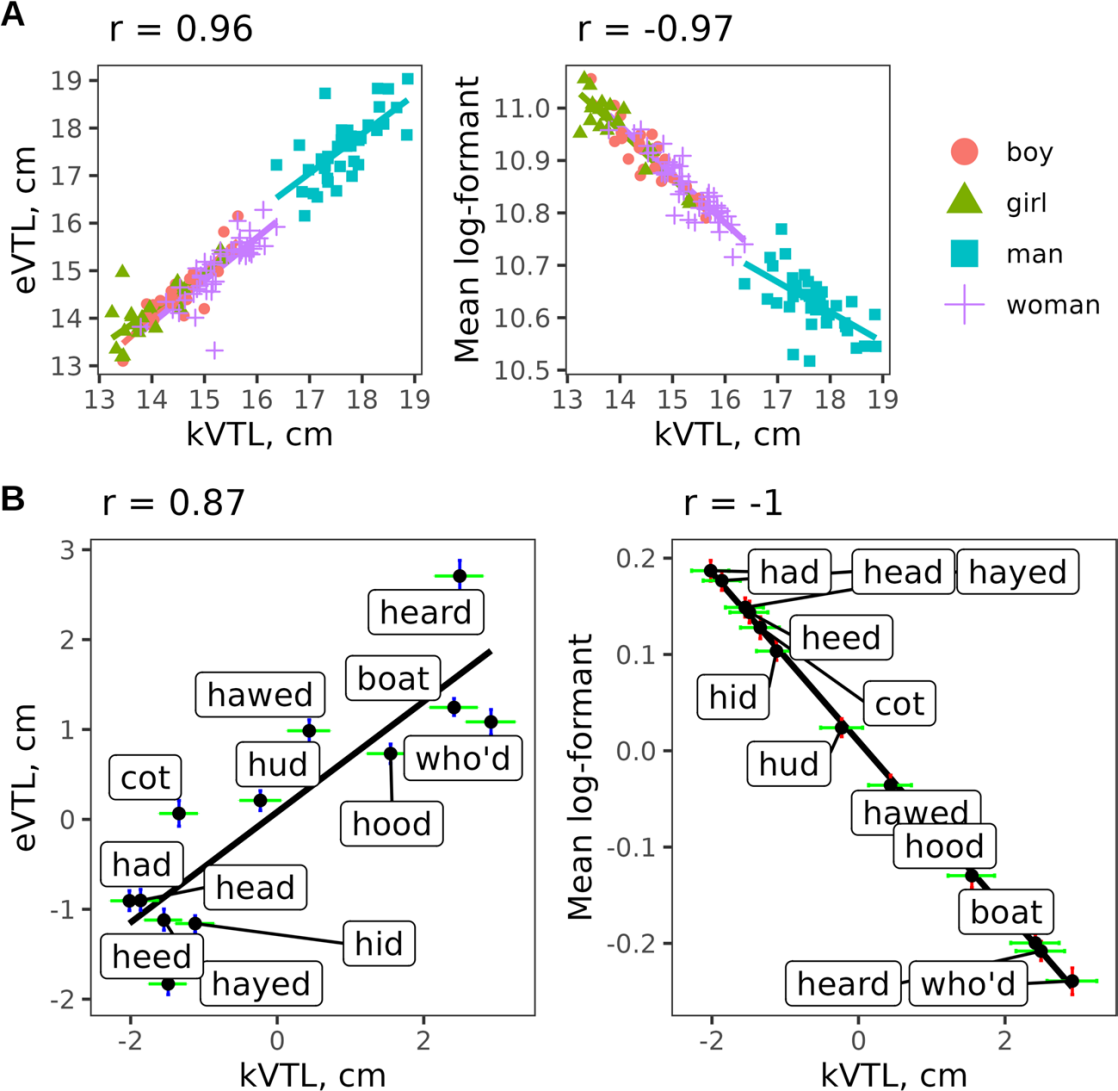
A comparison of scale estimates by three methods. Scatterplots and Pearson’s correlations between kVTL, eVTL, and mean log-formant frequency calculated (**A**) per speaker relative to mean eVTL and (**B**) per vowel relative to the mean across vowels in Hillenbrand et al. (1995). *Error bars* show 95% CIs around the estimate for each vowel

In the absence of anatomical measurements, we can only observe that all three multiple estimates of apparent VTL per speaker, which take into account up to 12 vowels from each speaker in Hillenbrand’s dataset, are quite similar: the scale factors that they predict correlate with Pearson’s *r* = .96 to .97 if we average across men, women, boys, and girls (Fig. 5A). Despite some differences between the algorithms, particularly when modeling the vocal tract of adult males, there is good agreement across the full range of human VTL values. Thus, in practice it should not matter very much which algorithm is used – as long as each speaker provides several vowels and the goal is to estimate speaker-specific VTL in relation to other speakers, not the anatomical ground truth.

Apart from speaker-specific VTL, model K1 can be used to calculate estimates of vowel-specific *k* (Fig. 5B). In other words, once we have accounted for differences in VTL between speakers and articulatory effects on the relative positions of each formant, for each vowel there remains a change in apparent VTL, which can be calculated as the main effect of *vowel* averaging across all formants (alternatively, we could focus only on the more stable upper formants; see supplementary code *model_K1.html* for implementation details). It is interesting to compare these estimates of the ‘size’ of different vowels derived from eVTL and mean log-formant methods. To do so, we can estimate the mean log-formant or eVTL per recording, then per speaker (averaging across all 12 vowels), and take the difference between them. Likewise, kVTL estimates per vowel can be extracted as the main effect of *vowel* from model K1.

As shown in Fig. 5B, kVTL residuals per vowel are nearly identical to mean log-frequency residuals, while eVTL estimates are somewhat different (*r* = .87). The most noticeable difference is that the low F3 in /ɝ/ makes its eVTL nearly 2 cm greater than that of /u/, whereas kVTL is slightly larger in /u/ than in /ɝ/. Thus, while all methods find systematic differences in VTL across vowels, the results are far from identical and difficult to verify in the absence of anatomical data, requiring validation in future studies. In general, when VTL is estimated using a single method and with similar ranges of vowel quality across all subjects (e.g., the same phrase or the same range of vowels), proportional differences in VTL estimates should relate to proportional differences in VTL between speakers. However, when these conditions are not met, the relationship between VTL estimates and actual VTL is substantially opaquer. Therefore, researchers are advised to be very cautious when interpreting differences in apparent VTL (regardless of how it is calculated) between animal or human vocalizations of variable vowel quality.

Given the considerable variation in apparent VTL across vowels, it is clearly preferable to record and analyze several different vowels if the anatomical relaxed-configuration VTL of a particular speaker is the measure of interest. Indeed, one-shot estimates of eVTL and mean log-formant correlate with kVTL with Pearson’s *r* = .70 and .62, respectively, which is quite a drop from *r* = .97 between kVTL and multiple eVTL estimates. In other words, the reproducibility of speaker-typical VTL estimates drops dramatically when only a single vowel is available, and it seems unlikely that the variation in eVTL across vowels precisely corresponds to the true changes in anatomical VTL as the same speaker pronounces different vowels. Of course, human nonverbal vocalizations and animal calls do not correspond to different vowels and tend to be schwa-like, so the variation of eVTL across vowels may be less of a problem outside speech, but this remains to be shown experimentally.

Finally, VTL is often of interest not for its own sake, but as a proxy measure for actual or perceived speaker size. Several commonly used summary measures, such as F4, mean formant, mean log-formant, and eVTL, are reported to be comparable predictors of actual speaker height, with the absolute value of Pearson’s correlations with height between .25 and .32 (Pisanski et al., 2014; Pisanski et al., 2016a). eVTL produced the highest correlation in the meta-analysis by Pisanski et al. (2014), and it has the advantage of being theoretically informed and linked to the constraints on voice production, so it is presumably a good choice if the actual anatomy of the vocal tract is of interest, but only as long as the recorded vowels or vocalizations are reasonably schwa-like. In future work, it will be interesting to test whether VTL estimates based on several tokens of different vowels offer a noticeable advantage over the traditionally used one-shot measures (Pisanski et al., 2014) for predicting speaker height.

How about the perceived, rather than actual, speaker size? It is well established that low and closely spaced formants create a powerful impression that the speaker is large (Barreda, 2016; Pisanski & Bryant, 2019), even though eVTL explains less than 10% of variance in actual height of adult humans (Pisanski et al., 2014). To demonstrate how well different acoustic estimates of VTL predict perceived speaker size, we re-analyzed the data from Barreda (2017a). In this study, consonant-vowel-consonant words with five different vowels were recorded as spoken by ten female speakers and then used in a perceptual rating study with the original or linearly scaled formants. The first six formants were measured (F1–F6). One-shot eVTL and mean log-formant correlated with perceived speaker height with Pearson’s *r* = 0.27 [0.25, 0.30] and 0.27 [0.24, 0.29], respectively. Crucially, scale estimates calculated from several tokens were much better predictors of perceived height: *r* = 0.36 [0.34, 0.38] for multiple eVTL, 0.41 [0.39, 0.44] for multiple mean log-formant, and 0.41 [0.39, 0.43] for *k*. The exact correlations are not so important – for example, in another dataset multiple eVTL came out on top as the best predictor of perceived vocal formidability (see supplements, *scenario3.html*). The key result here is that apparent VTL calculated from several tokens is consistently a much better predictor of perceived speaker size compared to estimates derived from a single token. It is as if human listeners perform vowel-adjusted size normalization from a single token, presumably by leveraging their extensive previous exposure to human speech (Turner et al., 2009; see Barreda, 2020 for an exploration of how this might work in perception).

How might we approximate this one-shot vowel adjustment performed by human listeners? One possibility is to categorize the vowel, assuming it is not known a priori (for example, from formant ratios), and then subtract the residual per vowel shown in Fig. 5B. For instance, if eVTL in a particular token is 15 cm and the vowel (based on formant ratios) is /i:/ as in /heed/, we add 1.8 cm to this eVTL, but if the vowel is / ɝ/, we subtract 2.7 cm. Barreda (2017a) reports that this simple correction results in ‘one shot’ estimates of average log-frequency that have a correlation of .81 with those obtained using the complete set of vowels. Nearey and Assmann (2007) also present a method that ‘guesses’ the vowel category and uses this to predict vowel quality and VTL with a high degree of accuracy using information from a single vowel token. Of course, these methods are only applicable when there is a closed set of possible sound categories to choose from, so they are not directly applicable to the general case where phonetic content, or the formant pattern more generally, may vary arbitrarily between speakers.

To conclude, listeners make some allowance for perceived vowel when asked to judge the size of speakers from a single token. This ability is not absolute: listeners still judge a speaker to be taller when they say /u/ rather than /ɑ/ (Barreda, 2017b). However, even this partial compensation for vowel is not easily matched by models that do not have access to several different vowels from the same speaker. In future it will be important to find a computational approach that better approximates human performance; for now, the bottom line is that it is advisable to obtain several vowels per speaker, or else to keep vowel quality or verbal content consistent across experimental conditions because it is very difficult to derive accurate estimates of speaker-typical VTL from a single vowel or to compare VTL across speakers and conditions when vowel quality is not controlled (see *scenario2.html* in supplements for an example). Provided that multiple vowels are recorded from each individual or that vowel quality is comparable across recordings, differences in VTL between individuals or experimental conditions can be captured using eVTL, mean log-formant, or scale factor *k*; all three methods should produce similar results.

## A comparison of methods for estimating vowel quality

**Fig. 6:**
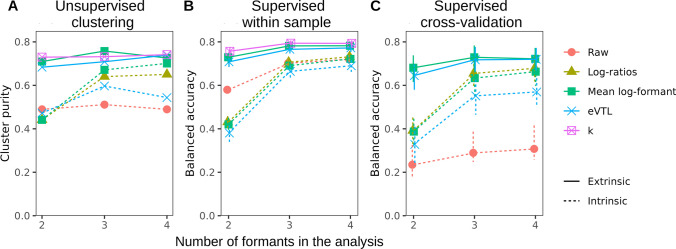
The effectiveness of vowel separation with different methods of formant normalization. **A** The purity of k-means clustering and **B** balanced accuracy (the average rate of true positives across the 12 vowels in Hillenbrand’s dataset) with Bayesian multi-logistic regression, either within the entire sample or **C** when training the model on children and testing it on adult men. The greatest improvement is achieved when also measuring upper formants (at least F3) and/or using token-extrinsic information. Raw = log-formants; log-ratios = musical intervals between consecutive formants; mean log-formant = subtract average of log-F1–F4; eVTL = calculate deviation from estimated neutral position with the *schwa* function; k = subtract scale factor per speaker estimated with mixed model K1. Intrinsic = using only a single token; extrinsic = using multiple token with different vowels from the same speaker. *Error bars* correspond to 95% CIs

Having compared the performance of different algorithms for estimating the length of a speaker’s vocal tract from formant frequencies, we now consider another aspect of speaker normalization, namely the extraction of scale-invariant formant patterns. The metric we use for this comparison is the degree of separation between different vowels in Hillenbrand’s set under different types of speaker normalization (Fig. 6), but the same principles apply when analyzing formant patterns in animals of greatly varying size such as adult males compared to infants of the same species. Johnson and Sjerps (2021) performed a similar comparison on a broader range of normalization methods and four datasets, obtaining the best results for extrinsic methods. We modified their procedure to make it more relevant to non-phonetic applications. First, for cross-species research it is crucial to test how well different normalization methods generalize beyond the VTL range in the training sample, which is here investigated by training a classifier on children’s voices in Hillenbrand’s dataset and testing it on adult men; the VTL ranges do not overlap at all between these groups (see *model_K1.html*). Second, to simulate research contexts in which it is not possible to measure upper formants (i.e., in quiet or noisy recordings), we also tested the performance of each algorithm when only some formants were available, namely F1–F2, F1–F3, or F1–F4. In addition to vowel classification accuracy in a supervised context, the performance was evaluated based on unsupervised clustering with k-means. There are many sophisticated metrics of clustering quality (Vinh et al., 2009), but in this case it can be expressed simply as the proportion of vowels that are assigned to the correct (majority-based) cluster, which we refer to as ‘cluster purity’, because the number of clusters is equal to the number of vowels.

Using intrinsic speaker normalization – that is, treating each token as if it were the only recording from each individual (dashed lines in Fig. 6) – the greatest improvement in both cluster purity and vowel classification accuracy is achieved by increasing the number of measured formants. F1 and F2 are not sufficient because the same F1–F2 combinations can correspond to different vowels depending on the (unknown) VTL. Interestingly, there is essentially no difference between raw and intrinsically normalized formant frequencies in terms of how well vowels can be recognized when working with the entire dataset and having F1–F3 or F1–F4 (dashed lines in Fig. 6B), as also reported by Johnson and Sjerps (2021). Observe, however, that raw formant frequencies are largely useless when training the model on children’s voices and then attempting to identify vowels spoken by adult men (Fig. 6C). In contrast, normalized measures show good transfer, only slipping a few percentage points when trained and tested within completely different VTL ranges. Interestingly, logarithmic methods for intrinsic normalization (formant log-ratios and mean log-formants) are somewhat better than intrinsic eVTL for vowel separation when extrapolating beyond the VTL range in the training sample.

Extrinsic speaker normalization – that is, pooling the information across multiple tokens per speaker, here up to 12 recorded vowels – leads to a noticeable improvement in vowel separation and classification (solid lines in Fig. 6). Multiple mean log-formant, eVTL, and *k* all produce very similar clustering quality and classification accuracy. Remarkably, and in marked contrast to intrinsic normalization, performance does not suffer much even when only F1 and F2 are measured. Overall, however, the availability of several recordings per speaker is not as essential for estimating VTL-normalized vowel quality as it is for estimating the actual or perceived speaker-typical VTL itself – provided that we also measure one or two higher formants in addition to F1–F2. Indeed, intrinsic normalization with any of the described methods should be precise enough for most purposes when it relies on all formants up to F4 or above.

## Sample applications

In this section we present several examples of research tasks in which the described algorithms can be used. The corresponding datasets and documented R code for each example are provided in the supplements, and here we merely highlight the main considerations that dictate the choice of analytic approach. The first step is always to locate relatively stable vowel-like regions in the recordings and measure average formants F1–F4 within each region. In this case, we used *formant_app* for manually correcting LPC estimates, as needed, and simply excluded all recordings that were too high-pitched or noisy to measure the formants reliably. While these particular recordings are by humans who were instructed to convey different attitudes (Anikin et al., 2023), vowel as a category is not used in any of the analyses, and precisely the same approach can be applied to animal vocalizations recorded in different contexts (e.g., agonistic vs. affiliative).

### Scenario 1

*Speakers were asked to sound either intimidating (large / strong / aggressive) or small / weak / submissive in different experimental conditions, and we are interested in whether speakers produce different formant patterns (vowels) depending on the condition.*

**Proposed solution** (*scenario1.html*). Since we have several recordings per speaker, we could perform extrinsic normalization by speaker-typical eVTL or mean log-formants from all available recordings from the same speaker. However, considering the very limited improvement over intrinsic normalization (Fig. 6B), we might as well simply analyze log-ratios (musical intervals) between formants or, for a more familiar and interpretable representation of the vowel space, calculate VTL-normalized formant frequencies using the *schwa* function. In this case, speakers trying to intimidate produced more open vowels, as indicated by the increase in speaker-normalized F1, which is presumably related to an attempt to vocalize more loudly.

### Scenario 2

*In the same experiment, we want to check whether speakers elongate their vocal tracts to intimidate.*

**Proposed solution** (*scenario2.html*). As we established above, different vowels are produced in different conditions, which immediately raises a red flag as any estimates of apparent VTL become suspect when the vowel quality is not controlled. To demonstrate that this is a very real concern, in this example we estimated differences in apparent VTL between experimental conditions using a wide variety of methods: single-shot or multiple eVTL and mean log-formant, model K1, and two more complex mixed models with *k* as a latent variable (model K2), including one that attempts to statistically correct for vowel quality when estimating scale factor *k*. All of these models agree that speakers shorten their vocal tract relative to baseline when they try to sound small / weak / submissive. However, eVTL estimates predict vocal tract elongation when speakers try to sound large / strong / aggressive relative to baseline, mean log-formant estimates predict vocal tract shortening, while mixed models K1 and K2 make predictions intermediate between these two extremes. The basic source of this mismatch is the different weighting of lower and upper formants: eVTL and related methods are less sensitive to vowel-related variation in formants F1 and F2. Even so, none of them are guaranteed to approximate true anatomical changes in VTL because any change in apparent VTL between conditions may be partly due to changes in formant patterns.

### Scenario 3

*We are interested in how formant frequencies are related to listeners’ ratings of formidability in a perceptual experiment.*

**Proposed solution** (*scenario3.html*). Both vocal tract elongation and changing formant patterns might contribute to conveying formidability, so we begin by estimating both, as in Scenarios 1 and 2. In this case, formidability is predicted by higher normalized F1 (an open vowel quality) and by all VTL measures, but especially by speaker-typical eVTL averaged across all produced tokens. Once again, this result confirms that listeners make allowance for vowel quality when judging speaker size from a single token, and the easiest way to model human performance is to “cheat” by using extrinsic normalization – that is, to pool information across multiple tokens and different vowels recorded from the same speaker. Which VTL measure is reported is a matter of personal preference and data availability: for example, eVTL is best for small datasets with missing values.

## Conclusion

The task of calculating vocal tract length and scale-invariant formant patterns in speech or nonverbal vocalizations can be thought of as the general case of vowel normalization in phonetics. Because it can be applied to a broad range of situations not involving a closed set of expected formant patterns, the general case of VTL and formant-pattern estimation brings its own specific objectives and methodological challenges. We have presented a comprehensive theoretical framework and toolkit for this work, and here we end with a quick summary, concrete guidelines and take-home points, as well as the most important gaps to address in future studies.

First of all, we would like to emphasize once again the crucial importance of verifying and, if necessary, correcting the automatic LPC estimates of formant frequencies. The freely available tools for this work include Praat (Boersma, 2006) and its plugins (Barreda, 2021a), and now also an interactive web app, *formant_app*, designed for quickly extracting manually verified average formant frequencies in entire vocalizations or annotated regions of interest. This step is no longer prohibitively time-consuming, and it can have a more dramatic impact on research conclusions than any of the subtleties of the following statistical analyses.

Formant analysis on a linear scale using the regression method (Reby et al., 2005; Reby & McComb, 2003) is based on the principle that the overall formant spacing in an approximately cylindrical tube depends on its length (VTL), while the pattern that these formants make (vowel quality) depends on the tube’s shape. Formant spacing dF can be estimated from formant frequencies in one or more tokens using simple linear regression with the *estimateVTL* function (resulting in what we call eVTL), and deviations from equal spacing normalized by dF are returned by the *schwa* function. This method is robust to missing formant values and applicable to vocalizations produced with the vocal tract in the configuration of a single, reasonably cylindrical tube, from human vowels to elephant rumbles. The lowest one or two formants have very little effect on eVTL estimates, so it is preferable to leave them blank if these formants are difficult to measure accurately (e.g., if LPC locks to harmonics in high-pitched calls), instead of analyzing incorrect values or dropping the entire vocalization from the analysis.

When seen on a logarithmic scale, formants form specific patterns or musical chords, which remain stable in speech sounds across speakers, being simply transposed up and down the frequency scale depending on the speaker’s VTL. The ratios or musical intervals between formants therefore constitute a scale-invariant representation of formant pattern, which we perceive as vowel quality in vowel-like sounds, while the mean log-formant gives a measure of overall scale. A more powerful statistical approach to this analysis is to estimate how far the ‘chords’ of each speaker are transposed relative to the average speaker in the sample using mixed models explained in the main text and in supplementary files (*model_K1.html*, *model_K2.html*, *scenario2.html*).

All these methods of VTL estimation produce comparable results when the task is to compare the typical VTL of each speaker averaging across the full range of vowels, as in Hillenbrand’s dataset. However, they make somewhat different predictions regarding the relation between vowel and VTL, and in the absence of suitable anatomical data it is difficult to validate these algorithms formally or to devise the optimal method of estimating speaker-typical, articulation-adjusted VTL from a single token (intrinsic normalization). Crucially, perceptual data indicate that listeners do adjust their size judgments depending on the perceived vowel, so that speaker-typical VTL estimates obtained with extrinsic normalization predict perceptual size ratings more successfully than do one-shot VTL estimates obtained with intrinsic normalization. Accordingly, if the research question concerns changes in the anatomical or perceived VTL in different conditions (e.g., in agonistic vs. affiliative contexts, when the speaker is trying to sound large or small, dominant, or submissive, etc.), it is advisable to record phonetically identical material in each condition: for example, a range of vowels or some standard phrase. Naturally, this is not an option when working with human nonverbal vocalizations or animal calls, and the development of anatomically and perceptually accurate methods for intrinsic normalization is an important area for further research.

The task is more straightforward when the formant pattern, rather than VTL, is the main object of interest. All of the described methods successfully capture the differences between human vowels, and they should be fully applicable to the task of describing vowel-like articulation in non-human animals of any size. There are only two simple rules to remember. First, non-normalized frequencies are not meaningful measures of the formant pattern when used across a wide range of VTLs, so some form of vocal tract normalization is nearly always required. Second, it is essential to measure at least one or two formants above F1–F2 to enable this normalization.

As a final note, LPC operates under the assumption that there are only positive resonances (poles), and VTL estimation with the regression method assumes that the vocal tract forms a single tube. It is therefore not applicable to calls produced with a more complex shape of the vocal tract – for example, to strongly nasalized vocalizations (Reby et al., 2016). However, there are two special cases that are amenable to the analysis methods presented in this paper. First, purely nasal vocalizations, such as some elephant rumbles (Beeck et al., 2022), can be analyzed under the assumptions of the closed-open tube model, where the vocal tract extends from the glottis to the nostrils. Second, single-tube (weakly nasalized) closed-mouth vocalizations might be analyzed with the closed-closed tube model, as shown in Figure 2C. However, extreme caution is needed, and we do not recommend comparing closed-mouth and/or nasalized vocalizations with non-nasalized calls in the context of VTL analysis because of the potential for making serious errors. Extending the existing tools for single-tube VTL estimation and vowel normalization to more acoustically complex resonators is another important avenue for future research.
